# Living Donor Liver Transplantation for Caroli's Disease: A Report of Two Cases

**DOI:** 10.5402/2011/106487

**Published:** 2011-04-27

**Authors:** Klaus Steinbrück, Marcelo Enne, Reinaldo Fernandes, Jose M. Martinho, Lúcio F. Pacheco-Moreira

**Affiliations:** Liver Transplantation Unit, Bonsucesso Federal Hospital, Health Ministry, Rio de Janeiro, 21041-030, RJ, Brazil

## Abstract

Caroli's disease (CD) is a rare autosomal recessive disorder characterized by intrahepatic cystic dilatation of the bile ducts. Patients with bilobar or progressive disease may require orthotopic liver transplantation (OLT). In the MELD era, living donor liver transplantation (LDLT) raised as the ultimate treatment option for these patients, once their MELD score is usually low. Herein, we describe 2 cases of patients (a 2-year-old girl and a 19-year-old teenager) that successfully underwent LDLT as a treatment for diffuse CD. The good postoperative courses of the two cases indicate that LDLT is a feasible option in the treatment of this disorder, even in complicated or early age patients.

## 1. Introduction

Caroli's disease (CD) is a rare autosomal recessive disorder characterized by intrahepatic cystic dilatation of the bile ducts. It was first described in 1906 [1] and named after Caroli in 1958 [2]. It is considered type V of Todani's classification for bile duct cysts [3]. Clinical progression and presentation of patients with CD is heterogeneous as long as symptoms may be absent for years or may occur at a very early age. When progressive, it leads to recurrent cholangitis, jaundice, right upper quadrant abdominal pain, intrahepatic stones accumulation, portal hypertension, and liver failure [4]. When combined with congenital hepatic fibrosis, it is known as Caroli's syndrome (CS) or Grumbach's disease. In this case, portal hypertension and hepatic fibrosis are responsible for the clinical manifestations. Many renal disorders have been described in association with these diseases, including autosomal recessive polycystic kidney disease [5].

Additionally, CD poses another life-threatening problem: it has a relatively high incidence of intrahepatic malignant tumors. Some authors have found cholangiocellular carcinoma (CCC) in 7% to 25% of patients, suggesting that the risk of malignant tumors developing is 100 times greater than that of the general population [6, 7].

Patients with bilobar disease with recurrent cholangitis or complications related to portal hypertension may require orthotopic liver transplantation (OLT). After the MELD score implantation, living donor liver transplantation (LDLT) raised as the ultimate treatment option for patients with CD complicated by recurrent cholangitis or portal hypertension, not amenable to conservative treatment. This is explained by the low score often achieved by these patients, as other cholestatic diseases. Few case reports have described experience of OLT in patients with CD, especially in children. CD is a rare indication for liver transplantation that accounts for 0.13% of all liver transplants performed in the United States [8]. Herein we describe 2 cases of patients that underwent LDLT as a treatment for CD. 

## 2. Material and Methods

**Figure 1:**
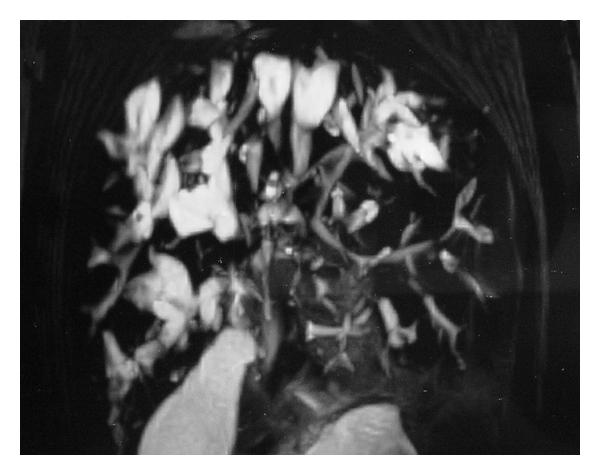
Preoperatory magnetic resonance of patient 1, showing the intrahepatic cystic dilatation of the bile ducts, especially on the right liver.

**Figure 2:**
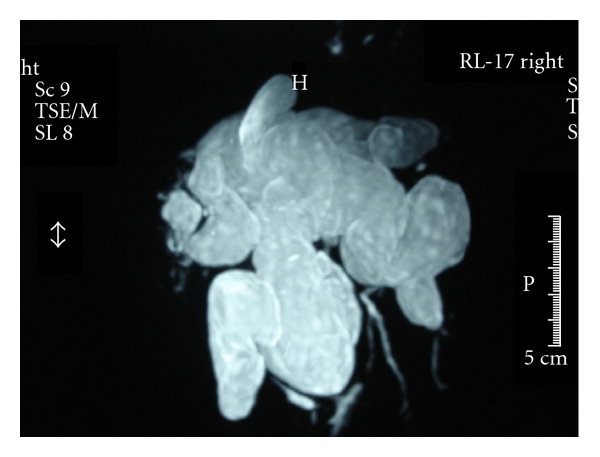
Preoperatory magnetic resonance of patient 2, reveling diffuse cystic dilatation of the bile ducts.

Between March 2002 and January 2011, a total of 400 liver transplants were performed at our liver transplantation unit. Among these, 142 received graft from a living donor. Two patients were transplanted due to diffuse CD. The diagnosis was based on clinical presentation, magnetic resonance image (Figures 1 and 2), and pathological findings.

**Case 1**

A 2-year-old girl, who was diagnosed with CD associated to cirrhosis when she was 6 months old due to symptomatic hepatomegaly. Her PELD score was 6. She was submitted to an LDLT and received a left lobe graft from her father. The graft weighted 240 g and corresponded to 1.96% GRWR. Surgery recovering was uneventfully for both patients. Donor and recipient were discharged on the 6th and 21th postoperative day (POD), respectively. Immunosuppression was achieved using Methylprednisolone and Tacrolimus. She is in good health after 82 months posttransplantation.

**Case 2**

**Figure 3:**
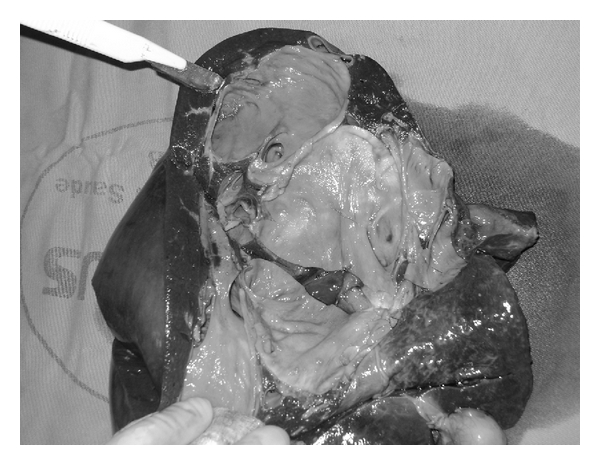
Inspection of liver specimen of patient 2 confirmed intrahepatic bile ducts' cystic dilatation.

A 19-year-old girl, who was diagnosed with bilobar CD when she was 13 years old, after an episode of cholangitis. Her MELD score was 15. The poor quality of live caused by many jaundice and cholangitis events led to transplantation. She underwent an LDLT and received a right liver graft from her father. The graft's weight was 706 g, corresponding to 1.21% GRWR. Liver specimen confirmed intrahepatic cystic dilatation of the bile ducts (Figure 3). Donor had an uneventful recovery and was discharged on the 7th POD. At the 10th POD, the recipient presented biliary complication, named bile leak with infected collection, which was submitted to percutaneous drainage and antibiotic treatment. No other complication occurred. She was discharged on the 29th POD and is well after 30 months of posttransplantation follow-up. Postoperatively, Tacrolimus and Methylprednisolone were prescribed as immunosuppressive agents.

In both cases, the recipient extrahepatic bile duct was resected and hepaticojejunostomy was performed. 

## 3. Discussion

CD is a rare congenital disorder characterized by multifocal segmental and communicating saccular or cystic dilatations of the intrahepatic bile ducts and is believed to be caused by an incomplete and faulty remodeling of the embryonic ductal plate [9]. There are two forms of the disease, one associated with congenital hepatic fibrosis, which is called CS, and the other a simple form occurring alone. The hereditary aspect of CD and CS is generally considered autosomal recessive.

The disease prevalence is 1/1,000,000 of the population, but with better image techniques, such as magnetic resonance cholangiography, which offer reliable pictures of the biliary system, CD appears to be more prevalent than previously reported [10].

Management of patients with CD or CS is still conflicted, because time and severity of onset can vary a lot. The long-term prognosis for these patients is poor, especially when recurrent cholangitis or extensive bilobar disease is present. Tsuchida et al. reviewed 50 cases reported since 1968 and identified 46% of mortality, primarily as a result of septicemia, liver abscesses, liver failure, and portal hypertension [11]. For patients with diffuse manifestations of CD, combined approaches with partial hepatectomy and biliodigestive anastomosis were not able to prevail. Even though extended resections may be suitable for a few patients, total replacement of the liver has been considered to be a more effective treatment [12]. Moreover, the high incidence of CCC associated with CD is another relevant issue. Dayton et al. presented 10 patients with CCC in CD who had a median age of 52 years and a maximum survival of 4 months, evidencing the threat of this complication [6]. On this scenario, liver transplantation emerged as a curative option for these patients. 

On a MEDLINE search, we identified 22 reports of liver transplantation for CD or CS [7–10, 12–29]. Information about the transplants (number, deceased donor or living donor, and follow-up time) and recipients (age, and survival for 1, 3, and 5 years) was reported on Table 1. 

Ulrich et al. showed a 100% survival after a nine-year followup for 4 patients submitted to OLT, from a deceased donor (DD), due to CD or CS [9]. De Kerckhove et al. presented data from the European Liver Transplantation Registry and noticed that 89 from 110 patients that underwent OLT to control CD or CS were still alive after a median follow-up of 812 days [23]. The large single-center report, analyzing 30 patients transplanted for CD or CS, was published by Habib et al., from Pittsburgh. Overall long-term graft survival rates at 1, 5, and 10 years were 73%, 62%, and 53%, and patient survival rates were 76%, 65%, and 56%, respectively, [12]. All patients had DD liver transplantation (DDLT). On a review carried out by Millwala et al., 104 patients with CD were identified from the UNOS Standard Transplant Analysis and Research files of liver transplant recipients between 1987 and 2006. CD or CS corresponded to 0.13% of all indications for OLT. LDLT was performed in 3.8% of these patients. The overall graft and patient survival at 1, 3, and 5 years were 79.9%, 72.4%, and 72.4%, and 86.3%, 78.4%, and 77%, respectively, [8]. Data show that liver transplantation for CD or CS is a secure procedure with an encouraging long-term outcome. Results are comparable to those who undergo OLT for other etiologies of chronic liver disease [11, 14, 28, 29].

A total of 174 patients were submitted to OLT for CD or CS, according to MEDLINE search. DDLT was carried out on 167 patients (96%). Seven patients (4%) underwent LDLT. Our two cases were submitted to LDLT, unlike the majority of patients reported. It is important to notice that in areas of the world where there is a crucial shortage of potential liver grafts, as occurs in Brazil with 7.2 donors/million habitants/year, [30] LDLT is the only option for patients with complicated CD, especially because of the low MELD score achieved by them. Isolated cases show no long-term outcome difference between LDLT and DDLT as a treatment for CD or CS [7, 22]. 

## 4. Conclusion

We successfully performed LDLT for 2 patients with diffuse Caroli's disease. The good postoperative courses of the two cases reported here indicate that LDLT is a feasible option in the treatment of this disorder, even when complicated by recurrent cholangitis or carried out in an early age patient. LDLT may be the only alternative for patients with complicated CD in regions with a lack of DD grafts. 

## Figures and Tables

**Table 1:** Liver transplantation for Caroli's Disease information researched on MEDLINE Database.

Author	Year	*N*	DDLT	LDLT	Age (years)	F/M	Kidney Tx	Suvival (1 y-3 y-5 y)	Followup (months)
Balsells [13]	1993	1	1	—	25	1F	—	100%-NA-NA	24
Shiano [14]	1997	1	1	—	35	1F	—	100%-NA-NA	21
Sans [15]	1997	2	2	—	41.5 (21–62)	1F/1M	—	NA-NA-NA	36 (8–64)
Marx [16]	1999	1	1	—	24	F	—	NA-NA-NA	3
Waechter [17]	2001	2	2	—	36.5 (32–41)	2M	—	NA	NA
Takatsuki [18]	2001	1	—	1	36	1F	—	100%-NA-NA	29
Ammori [19]	2002	5	5	—	NA	NA	1/5	80%-NA-NA	20 (15–32)
Ninan [20]	2002	1	1	—	25	1F	—	100%-100%-NA	48
Madjov [21]	2005	1	1	—	25	1M	—	NA-NA-NA	NA
Kassahun [10]	2005	2	2	—	NA	2F	—	100%-100%-NA	48
Dalgic [22]	2005	1	—	1	NA	NA	—	NA-NA-NA	NA
De Kerckhove [23]	2006	3	3	—	38.3 (11–68.1)	1F/2M	2/3	NA-NA-NA	98.6 m (8–204)
Bockhorn [7]	2006	2	1	1	NA	NA	—	100%-NA-NA	31
Habib [12]	2006	30	30	—	25.5	15F/15M	—	76%-NA-65%	91
Mabrut [24]	2007	5	5	—	NA	NA	2/5	NA-NA-NA	NA
Liu [25]	2007	2	2	—	NA	NA	—	100%-100%-0%	41.5 (36–47)
Millwala [8]	2008	104	100	4	35.1	57F/47M	8/104	86.3%-78.4%-77%	NA
Amezaga [26]	2008	1	1	—	45	NA	—	NA-NA-NA	NA
Wang [27]	2008	3	3	—	15.3 (12–33)	2F/1M	—	100%-66%-NA	54.7 (34–82)
Ulrich [9]	2008	4	4	—	42.7 (26–60)	2F/2M	1/4	100%-100%-100%	109 (70–148)
Meier [28]	2008	1	1	—	8.2	1F	—	100%-100%-NA	57
Aguilar [29]	2008	1	1	—	44	1F	—	100%-100%-100%	72

DDLT: deceased donor liver transplantation; LDLT: living donor liver transplantation; F/M: female/male; NA: not available.
